# Higher and Sustained Cell-Mediated Immune Responses After 3 Doses of mRNA COVID-19 Vaccine in Patients With Inflammatory Bowel Disease on Anti–Tumor Necrosis Factor Therapy

**DOI:** 10.14309/ctg.0000000000000688

**Published:** 2024-04-25

**Authors:** Freddy Caldera, Stacey Rolak, Francis A. Farraye, Brian M. Necela, Davitte Cogen, Emily E. Zona, Trevor L. Schell, Oscar Ramirez Ramirez, Mazen Almasry, Kelly Chun, Mary S. Hayney, Keith L. Knutson

**Affiliations:** 1 Department of Medicine, Division of Gastroenterology and Hepatology, University of Wisconsin School of Medicine & Public Health, Madison, Wisconsin, USA;; 2 Department of Medicine, Mayo Clinic, Rochester, Minnesota, USA;; 3 Inflammatory Bowel Disease Center, Department of Gastroenterology and Hepatology, Mayo Clinic, Jacksonville, Florida, USA;; 4 Department of Immunology, Mayo Clinic, Jacksonville, Florida, USA;; 5 University of Wisconsin School of Medicine and Public Health, Madison, WI;; 6 Department of Internal Medicine, University of Wisconsin School of Medicine and Public Health, Madison, Wisconsin, USA;; 7 LabCorp, R&D and Specialty Medicine, Calabasas, CA, USA;; 8 School of Pharmacy, University of Wisconsin School of Medicine & Public Health, Madison, Wisconsin, USA.

**Keywords:** Crohn's disease, ulcerative colitis, immunology, immune response

## Abstract

**INTRODUCTION:**

Studies suggest that the generation of durable T-cell immunity following coronavirus disease 2019 (COVID-19) vaccination protects against severe disease. The aim of this study was to measure cell-mediated immune response (CMIR) 1–2 months and 6 months after a third dose of a COVID-19 mRNA vaccine.

**METHODS:**

This prospective study (HumoRal and CellULar initial and Sustained immunogenicity in patients with inflammatory bowel disease [IBD]) evaluated CMIR at 28–65 days (t_1_) after dose 2, 28–65 days (t_2_) (n = 183) and 6 months (±45 days) (t_3_) (n = 167) after a third dose of an mRNA COVID-19 vaccine. A small cohort had blood sample available 28–65 days (t_4_) (n = 55) after a fourth dose. Primary outcomes were CMIR at (t_2_) and (t_3_). Secondary outcomes included the effect of immunosuppressing IBD medications on CMIR and response at (t_4_).

**RESULTS:**

All patients had measurable CMIR at all time points. CMIR increased at t_2_ compared with that at t_1_ (median 1,467 responding cells per million (interquartile range [IQR] 410–5,971) vs 313 (94–960) *P* < 0.001). There was no significant waning in t_2_ vs t_3_ or significant boosting at t_4_. Those on anti–tumor necrosis factor monotherapy had a higher CMIR compared with those not on this therapy at t_2_ (4,132 [IQR 1,136–8,795] vs 869 [IQR 343–3,221] *P* < 0.001) and t_3_ (2,843 [IQR 596–6,459] vs 654 [IQR 143–2,067] *P* < 0.001). In univariable analysis, anti–tumor necrosis factor monotherapy was associated with a higher CMIR at t_2_ (*P* < 0.001) and t_3_ (*P* < 0.001) and confirmed in a multivariable model (*P* < 0.001).

**DISCUSSION:**

A third dose of a COVID-19 vaccine boosts CMIR, and the response is sustained in patients with IBD.

## Graphical abstract

**Figure:**
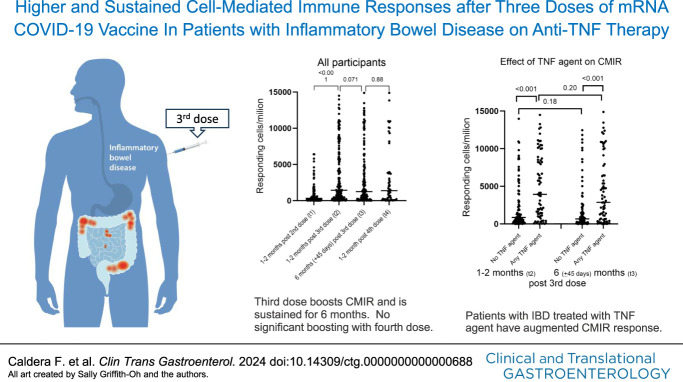

## INTRODUCTION

Four coronavirus disease 2019 (COVID-19) vaccines, mRNA-1273 (Moderna), BNT162b2 (Pfizer-BioNTech), Ad.26.COV2.S (Johnson & Johnson; no longer available in the United States), and NVX-CoV2373 (Novavax) have been found to be highly effective in the general population (1,2). The original clinical trials evaluating the efficacy of these vaccines excluded patients with inflammatory bowel disease (IBD) and other immunosuppressed populations, who historically may have a lower immune response to non–COVID-19 vaccines (3,4). COVID-19 vaccines have been found to be safe in patients with IBD with similar rates of localized and systemic adverse events as found in the general population (5,6). In addition, COVID-19 vaccination is not associated with IBD flares with low rates reported in prospective studies (6).

Multiple studies have evaluated humoral immunogenicity of COVID-19 vaccines and found that most patients with IBD are able to mount measurable antibody responses after the 2-dose mRNA vaccine series (95%–99%) (7–12). Robust humoral responses are seen after 3 doses with greater than 99% response rate, which are higher rates of seroconversion than that seen in other immunosuppressed populations such as solid organ transplant recipients (13–15). Furthermore, patients with IBD have a sustained humoral immune response to COVID-19 vaccines with most patients having measurable antibodies 6 months after a third dose of a COVID-19 vaccine (16). However, those on anti–tumor necrosis factor (TNF) therapy may have lower antibody concentrations after 2 or 3 doses of COVID-19 vaccines, with faster waning of antibodies (12). Those with waning humoral immunity may be more susceptible to breakthrough infections and boosting by additional doses are protective (17). While antibody concentrations wane with time after vaccination, cellular immunity may persist (18). SARS-CoV-2–specific cellular immune responses provide robust memory, mediate recognition of viral variants, and are important for viral clearance (18). In addition, many viral variants of concern may evade humoral immunity, but cellular responses induced by vaccines show strong protection against these variants (19). Thus, evaluating the COVID-19 vaccine–induced cell-mediated immune response (CMIR) in patients with IBD on immune-modifying therapy is of utmost importance.

In contrast to vaccine-induced humoral immunity, fewer studies have evaluated vaccine-induced CMIR in patients with IBD. Initial studies found that most patients develop CMIR after the mRNA primary series (9,20,21). Two studies found that those on anti-TNF therapy have a higher CMIR compared with those on non-TNF biologics or immunomodulators after 2 doses of vaccine (20,21). Two studies that evaluated CMIR immediately after a third dose of COVID-19 vaccine found that those on anti-TNF therapy mounted similar (10) or lower CMIR (22) compared with healthy controls. To further evaluate the CMIR after COVID-19 vaccination, the primary aim of this study was to measure CMIR at 1–2 months and approximately 6 months after a third dose of mRNA COVID-19 vaccine. Secondary aims were to evaluate the impact of a fourth dose; impact of immune-modifying therapies; effects of homologous or heterologous boosting; and prior COVID-19 infection on CMIR. We also aimed to examine the correlation between CMIR and humoral immune responses. We hypothesized that those on anti-TNF therapy would continue to demonstrate higher CMIR compared with patients with IBD on other treatments, as we have previously seen in our HumoRal and CellULar initial and Sustained immunogenicity in patients with IBD (HERCULES) cohort.

## METHODS

### Study design

The HERCULES study is a prospective study evaluating humoral responses and CMIR of COVID-19 vaccines in patients with IBD. Participants with IBD were enrolled at the University of Wisconsin–Madison. Methods and inclusion criteria for HERCULES have been previously described (8). In brief, eligibility criteria were a diagnosis of IBD, ages 18–85 years, on stable doses of maintenance therapy at least 2 months, and 3 mRNA vaccine doses. Patients were divided into the following groups: (i) The nonsystemic immunosuppressive group: on mesalamine monotherapy or no therapy for IBD or on vedolizumab monotherapy. Vedolizumab was considered in this group because previous studies have shown that it does not seem to affect vaccine response (4,23). (ii) The immunosuppressed group included the following: thiopurine therapy group: on azathioprine at least 2 mg/kg or 6 MP 1 mg/kg; anti-TNF therapy group: on maintenance therapy infliximab (at least every 8 weeks), golimumab (at least monthly), adalimumab (at least every 2 weeks), or certolizumab (at least monthly); anti-TNF combination therapy group: on anti-TNF therapy as described earlier along with either 15 mg of methotrexate or azathioprine at least 1 mg/kg or 6 MP 0.5 mg/kg; ustekinumab therapy group: on either ustekinumab monotherapy or combination therapy with methotrexate or azathioprine; tofacitinib therapy group: on tofacitinib at least 5 mg PO BID; and corticosteroid therapy group: on any one of the systemic immunosuppressive groups and any dose of corticosteroids.

We previously reported the CMIR at 28–65 days (t_1_) after the 2 dose primary series (21). In this study, we analyzed CMIR in lymphocytes available at 28–65 days (t_2_) and approximately 6 months (±45 days) (t_3_) after a third dose of an mRNA COVID-19 vaccine. A small cohort had blood sample available 28–65 days (t_4_) after a fourth dose of an mRNA vaccine. Samples were analyzed from individuals who were not lost to follow-up from the original cohort, had not changed treatment since the initial 2 dose series, and had received a third or fourth dose of a COVID-19 vaccine. Change in treatment was defined as changing in treatment group (e.g., from anti-TNF therapy to ustekinumab therapy). A change in dose or addition of an immunomodulator to a participant on a biologic was not considered a change in therapy. Participants who changed therapy between t2 and t3 were excluded.

Completion of an mRNA vaccines series was confirmed by review of the Wisconsin Immunization Registry (WIR). A history of COVID-19 infection was defined by a molecular-confirmed diagnosis of SARS-CoV-2 infection through polymerase chain reaction (PCR) confirmed in chart review or nucleocapsid antibody seroconversion after 28–65 days (t_2_) or approximately 6 months (±45 days) (t_3_) after a third dose. All patients signed informed consent. The study received instituational review board (IRB) approval at the University of Wisconsin-Madison School of Medicine and Public Health.

### Wisconsin Immunization Registry

The WIR is a state-wide database maintained by the Department of Health and Family Services of the State of Wisconsin in which vaccine data for each Wisconsin resident is stored. The WIR captures 97% of vaccines administered in the state, and 98.5% of Wisconsin residents have an active WIR record. The WIR does not capture vaccines administered outside the state, and all Wisconsin vaccine providers are required to enter the record of COVID-19 vaccine administration into the registry (24). The WIR has been previously used to evaluate COVID-19 vaccine uptake in patients with IBD (25).

### Outcomes

The primary outcomes of this study were CMIR at 1–2 months (28–65 days) (t_2_) and approximately 6 months (±45 days) (t_3_) after a third dose of an mRNA COVID-19 vaccine. We chose these time points to evaluate CMIR immediately after immunization (t_2_), the same time point used when we evaluated humoral and CMIR after a 2-dose series and sustained immunogenicity at 6 months because that is the typical time that has been used to evaluate sustained immunogenicity of vaccines in other studies (4,26).

Secondary outcomes included CMIR at 28–65 days (t_4_) after a fourth dose, evaluating the effects of homologous boosting (receiving same mRNA vaccine as primary series) when compared with heterologous boosting (different from the primary vaccine), effects of immune-modifying therapies on CMIR, particularly anti-TNF monotherapy, anti-TNF combination therapy with an immunomodulator, and history of COVID-19 infection (as defined earlier), and correlations between CMIR and humoral immune responses.

### Humoral immune response measurement

Nucleocapsid and spike protein S1 receptor binding domain–specific antibodies were measured in sera at 28–65 days (t_2_) and approximately 6 months (±45 days) (t_3_) after a third dose of an mRNA COVID-19 vaccine and at 28–65 days (t_4_) after a fourth dose. Specific antibodies measured in sera using electrochemiluminescent immunoassays performed at LabCorp were antinucleocapsid and anti-spike S1 receptor–binding domain–specific IgG antibodies reported as U/mL and mcg/mL respectively, as previously described at every time point (8).

### CMIR measurements

Fluorospot assays were performed to quantitate antigen-specific T cells capable of secreting interferon (IFN)–γ with use of the human IFN-γ FluorospotPlus kit (Mabtech) from samples at (t_2_), (t_3_), and at (t_4_), as previously described (21). Antigen-specific T cells were enumerated using spike protein peptide pools 1 + 2 (Stemcell Technologies, 1 μg/mL), which are pools of 158 peptides each consisting of 15-mer peptides with 11-amino acid overlaps that span amino acids 1–1,273 of the spike protein.

### Data collection

The following clinical variables were collected for participants. At the baseline visit, demographic data including age, gender, race, ethnicity, IBD diagnosis and treatment, and COVID-19 vaccine history. At the subsequent visits, patient interview and medical record review were performed to look for medication changes and COVID-19 vaccine history.

### Statistical analysis

CMIR between groups and time points were compared using Mann-Whitney U and Wilcoxon signed rank tests, respectively. Univariable and multivariable linear regression analyses were conducted to assess the association of CMIR at 28–65 days after dose 3 (t_2_) and at approximately 6 months (±45 days) after dose 3 (t_3_) with age, sex, mRNA vaccine type, homologous or heterologous boosting, previous COVID infection, and IBD therapy (aminosalicylate monotherapy, thiopurine monotherapy, anti-TNF therapy, vedolizumab, ustekinumab, systemic corticosteroids, or tofacitinib). To evaluate the association between CMIR and these variables, we used a multivariable linear regression model with variable that had a *P* value < 0.05. The residuals had constant variance and were normally distributed. Multivariable linear regression was performed to estimate the relationship among age, anti-TNF therapy, aminosalicylates, and CMIR. All tests were 2-sided with *P* value < 0.05 considered statistically significant. All analyses were performed using SPSS V27 or R studio 4.2.1.

## RESULTS

**Figure 1.**
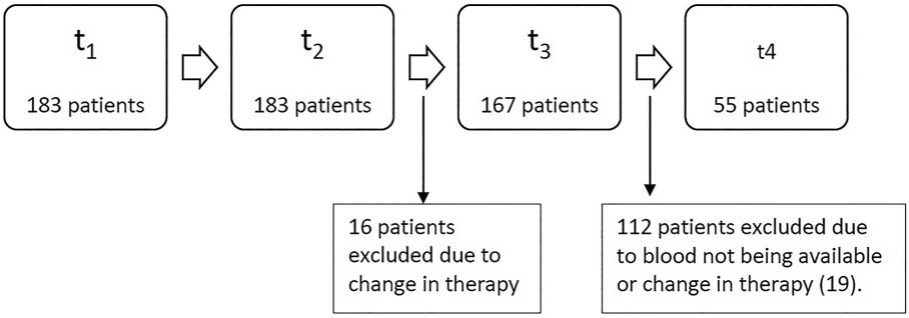
Flowchart of study participants in HERCULES study. Flowchart of participants in the HERCULES study at the different time points t_1_-t_4_. Blood draw timings: (**a**) (t_1_) 28–35 days after 2 dose mRNA vaccine series; (**b**) (t_2_) 28–65 days after a third dose; (**c**) (t_3_) approximately 6 months (±45 days) after a third dose of an mRNA COVID-19 vaccine; (**d**) (t_4_) 28–65 days after a fourth dose of an mRNA vaccine. HERCULES, HumoRal and CellULar initial and Sustained immunogenicity in patients with inflammatory bowel disease.

One hundred eighty-three patients had CMIR measured at 28–65 days after dose 3 (t_2_), 167 at approximately 6 months (±45 days) after dose 3 (t_3_), and 55 at 28–65 days after a fourth dose (t_4_) (Table 1 and Figure 1). The median time blood sample was collected at 28–65 days after dose 3 (t_2_) was 42 days (interquartile range [IQR] 35–51), and the median at approximately 6 months (±45 days) after dose 3 (t_3_) of blood collection was 168 days (IQR 147–175). Most of the individuals (169/183, 92%) received a homologous third dose that was the same as their original 2-dose primary series. Most of the individuals who received a fourth dose (52/55, 95%) also received the same as the primary series (Table 1). We previously reported that 89% of individuals at 28–65 days (t_1_) after dose 2 had a CMIR (20). All patients after 3 doses at (t_2_) and (t_3_) and after a fourth dose (t_4_) had a measurable CMIR. Thirty-eight (23%) individuals at approximately 6 months (±45 days) after dose 3 (t_3_) had a previous COVID-19 infection compared with 16 (9%) at 28–65 days after the third dose (*P* < 0.001).

**Table 1.** Demographics of patients with IBD

	28–65 d after third mRNA dose 3 (t_2_) n (%)	Six mo after third mRNA dose (t_3_) n (%)	After fourth dose (t_4_)
Total no. of patients	183	167	55
Age, yr	44 (35–59)	44 (35–59)	54 (40–64)
Male	88 (48%)	85 (51%)	31 (56%)
Non-Hispanic White	169 (92%)	152(91%)	50 (91%)
Ethnicity Hispanic	2 (1%)	2 (1%)	0
Crohn's disease	113 (61%)	101 (60%)	31 (56%)
Vaccine preparation			
All BNT162b2	86 (47%)	84 (57%)	—
All mRNA-1273	83 (45%)	71 (43%)	—
BNT162b2 ×2 then mRNA-1273	2 (1%)	2 (1%)	—
mRNA-1273 ×2 then BNT162b2	12 (7%)	10 (6%)	—
Heterologous 4th dose	—	—	3 (6%)
Previous COVID-19 infection	16 (9%)	38 (23%)	—
IBD medication			
Aminosalicylate or no IBD therapy	29 (16%)	29 (17%)	6 (11%)
Thiopurine monotherapy	14 (8%)	13 (8%)	5 (9%)
Systemic steroids (5–20 mg)	9 (5%)	12 (7%)	1 (2%)
Anti-TNF monotherapy	57 (31%)	50 (30%)	16 (29%)
Anti-TNF combination therapy^a^	22 (12%)	21 (13%)	0.85
Ustekinumab monotherapy or combination therapy^a^	23 (13%)	21 (13%)	11 (20%)
Vedolizumab monotherapy	33 (18%)	28 (17%)	11 (20%)
Tofacitinib	5 (3%)	5 (3%)	0

Values are median (interquartile range) or n (%).

IBD, inflammatory bowel disease; TNF, tumor necrosis factor α.

a Including azathioprine, 6-mercaptopurine, or methotrexate.

### Cell mediated immune response post third dose (t_2_ and t_3_)

**Figure 2.**
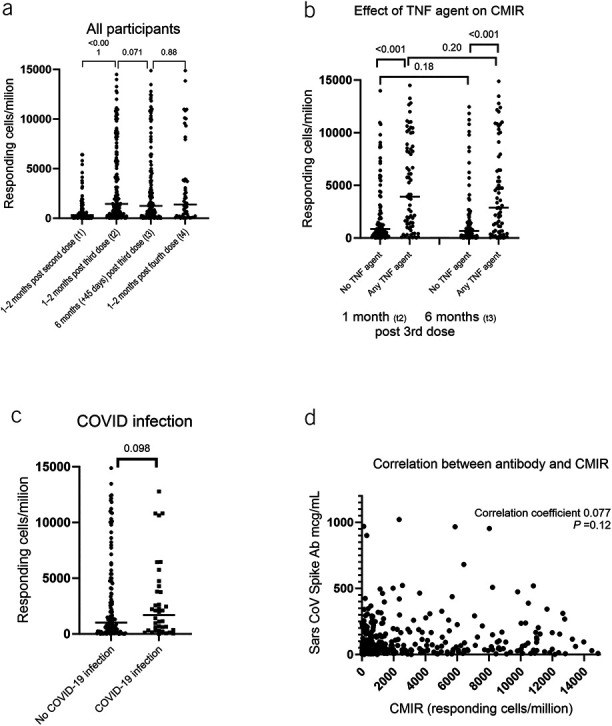
Cell-mediated immune response (CMIR) following COVID-19 mRNA vaccines. (**a**) CMIR increased after the 3rd vaccine dose (n = 161; median 1,444 responding cells/million (interquartile range [IQR] 421–6,011) compared with after 2 vaccine doses (n = 120; median 313 responding cells/million; IQR 94–960) *P* < 0.001. The CMIR approximately 6 months after the 3rd dose was not statistically significantly different from 1 month after dose 3 (median 1,241 responding cells/million (IQR 301–4,609) Wilcoxon signed rank *P* = 0.071). The CMIR did not boost following the 4th dose of vaccine (n = 55; median 1,387 responding cells/million [IQR 203–3,843] *P* = 0.88). (**b**) Patients with IBD treated with TNF agents (n = 69) had higher CMIR compared with those not treated with TNF agents (n = 92) at both 28–65 days after 3rd dose (median 3,927 responding cells/million [IQR 1,094–8,619] vs median 853 responding cells/million [IQR 343–2,984] *P* < 0.001) and at approximately 6 months post 3rd dose (median 2,876 responding cells/million [IQR 614–7,094] vs median 671 responding cells/million [IQR 161–2,191] *P* < 0.001). In addition, neither group showed statistically significant waning of CMIR over 6 months. (**c**) History of COVID-19 infection had no statistically significant effect on CMIR at 6 months after dose 3 (no COVID-19 infection median 1,024 responding cells/million; IQR 228–4,609 vs COVID-19 infection history median 1,690 responding cells/million; IQR 311–3,913; *P* = 0.098). (**d**) No statistically significant correlation was found between CMIR and SARS CoV spike antibody (Ab) concentrations (Spearman correlation coefficient 0.077; *P* = 0.12). This graph shows data points from all time points post vaccines. CMIR-antibody correlations for each time point are included in the Supplementary Digital Content (see Supplementary Figure 1, http://links.lww.com/CTG/B89). TNF, tumor necrosis factor.

CMIR increased at (t_2_) compared with after the primary 2-dose series (t_1_) (t_2_ median 1,444 responding cells/million [IQR] 421–6,011) vs t_1_ median 313 responding cells/million [94–960] *P* < 0.001 (8)) (Figure 2a). Waning of CMIR over the 6-month interval following dose 3 was not statistically significant (n = 161 pairs with samples at both time points; t_3_ median 1,241 cells/million [IQR 301–4,609] Wilcoxon signed rank *P* = 0.071). No significant boosting was seen at t_4_ (t_4_ median 1,387 responding cells/million [IQR 203–3,843] *P* = 0.88). Those on anti-TNF monotherapy had a higher CMIR compared with those not on this therapy at t_2_ (median 3,927 [IQR 1,094–8,619] vs 853 [IQR 343–2,984] Mann-Whitney *U* test *P* < 0.001) and t_3_ (median 2,876 [IQR 614–7,094] vs 671 [IQR 161–2,191] *P* < 0.001). CMIR waning was not statistically significant in either the TNF agent group (t_2_ vs t_3_ Wilcoxon signed rank *P* = 0.20) nor the non–TNF agent group (t_2_ vs t_3_ *P* = 0.18) (Figure 2b). At (t_3_), history of COVID-19 infection did not affect CMIR (no COVID-19 infection median 1,024 responding cells/million; IQR 228–4,609 vs COVID-19 infection history median 1,690 responding cells/million; IQR 311–3,913; *P* = 0.098) (Figure 2c). No correlation between antibody concentrations and CMIR was found at any time point (Figure 2d and see Supplementary Figure 1, http://links.lww.com/CTG/B89). The number of participants who received heterologous vaccine series was insufficient to test the hypothesis that CMIR would be different between homologous and heterologous series (homologous series: n = 155; median 1,271 responding cells/million; IQR 305–4,280 vs heterologous series: n = 12; median 472 responding cells/million; IQR 56–5,253; *P* = 0.30).

### Univariable and multivariable analyses at 28–65 days post third dose (t_2_)

In univariable analysis evaluating CMIR at (t_2_), increasing age (beta coefficient (β = −51.16, *P* = 0.009), being on aminosalicylate therapy (β = −1,517.9, *P* = 0.046), or being on systemic corticosteroids (β = −2,805.9, *P* = 0.035) was associated with a lower CMIR. Being male (β = 1,199.4, *P* = 0.037) and being on anti-TNF monotherapy were associated with a higher CMIR (β = 2,627.4, *P* < 0.001). Those on anti-TNF combination therapy did not have higher or lower CMIR (Table 3). In our multivariable model, we confirmed that being on anti-TNF monotherapy was associated with a higher CMIR (β = 2,056.9, *P* < 0.001). Being male was also associated with higher CMIR (β = 1,152.4, *P* < 0.036). No other variables were associated higher or lower CMIR in the multivariable model (Table 2).

**Table 2.** Multivariable models evaluating cell-mediated immune response 28–65 days after a third dose of a COVID-19 vaccine (N = 183)

	Univariable			Multivariable		
	Beta coefficient	95% confidence interval (CI)	*P* value	Beta coefficient	95% CI	*P* value
Age (continuous variable)	−51.16	−89.4 to −12.9	0.009	−32.4	−70.4 to 5.6	0.09
Male	1,199.4	73.1 to 2,325.7	0.037	1,152.4	77.2 to 2,227.5	0.036
mRNA vaccine type (mRNA-1273)	−25.2	−1,165.1 to 1,114.8	0.965			
Type of boosting (heterologous) (n = 14)	−348.2	−2,940.4 to 1,794	0.749			
Previous COVID-19 infection (n = 16)	−1,074.6	−3,084.8 to 935.6	0.293			
IBD medication						
Aminosalicylate or no IBD therapy (n = 29)	−1,571.9	−3,114.5 to −29.4	0.046	−675	−2,221.1 to 869.5	0.389
Thiopurine monotherapy (n = 14)	−1,879.5	−4,004.2 to 245.6	0.083			
Systemic corticosteroids (n = 9)	−2,805.9	−5,407.4 to −204.3	0.035	−1,927.7	−4,482.6 to 639.2	0.14
Anti-TNF monotherapy (n = 57)	2,627.4	1,459.3 to 3,795.3	<0.001	2,056.9	826.3 to 3,287.5	<0.001
Anti-TNF combination therapy^a^ (n = 22)	939.2	−806.7 to 2,685.1	0.29			
Vedolizumab monotherapy (n = 33)	−1,207.7	−2,678.5 to 263.1	0.107			
Ustekinumab monotherapy or combination^a^ (n = 23)	−1,601.8	−3,303.9 to 100.2	0.065			
						
Tofacitinib (n = 5)	1,284.9	−2,203.8 to 4,773.7	0.468			

bold entries meant the values were statistically significant.

CI, confidence interval; COVID-19, corona virus disease 2019; IBD, inflammatory bowel disease; TNF, tumor necrosis factor α.

a Including azathioprine, 6-mercaptopurine, or methotrexate.

### Univariable and multivariable analyses at approximately 6 month post third dose (t_3_)

In univariable analysis evaluating CMIR at (t_3_), increasing age (beta coefficient (β = −46.5, *P* = 0.02) or being on aminosalicylate therapy (β = −1,752.8, *P* = 0.024) was associated with a lower CMIR, while being on anti-TNF monotherapy was associated with a higher CMIR (β = 2,469.9, *P* < 0.001). Those on anti-TNF combination therapy did not have higher or lower CMIR (Table 3). In our multivariable model, we confirmed that being on anti-TNF monotherapy was associated with a higher CMIR (β = 2,089.3, *P* < 0.001). No other variables were associated with a lower or higher CMIR in the multivariable model (Table 3).

**Table 3.** Multivariable models evaluating cell-mediated immune response approximately 6 months (±45 days) after third mRNA dose (t_3_) (n = 167)

	Univariable			Multivariable		
	Beta coefficient	95% CI	*P* value	Beta coefficient	95% CI	*P* value
Age (continuous variable)	−46.5	−85.6 to −7.4	0.0199	−27	−66.3 to 12.3	0.176
Male	783.4	−363.8 to 1,930.6	0.179			
mRNA vaccine type (mRNA-1273)	405.7	−746.4 to 1,557.9	0.488			
Type of boosting (heterologous) (n = 12)	−188	−2,421.1 to 2,044.3	0.868			
Previous COVID-19 infection (n = 38)	106	−1,269.3 to 1,481.3	0.879			
IBD medication						
Aminosalicylate or no IBD therapy (n = 29)	−1,725.8	−3,224.7 to −226.8	0.0243	−738.6	−2,275.3 to 789.1	0.344
Thiopurine monotherapy (n = 13)	−1,308.9	−3,451.8 to 833.9	0.23			
Systemic corticosteroids (n = 12)	−404.6	−5,347.3 to 1,392.6	0.721			
Anti-TNF monotherapy (n = 50)	2,469.9	1,269.3 to 3,670.3	<0.001	2,089.3	815.7 to 3,362.8	<0.001
Anti-TNF combination therapy^a^ (n = 21)	652.8	−1,083.5 to 2,389	0.459			
Vedolizumab monotherapy (n = 28)	−1,350	−2,879.6 to 179.6	0.083			
Ustekinumab monotherapy or combination^a^ (n = 21)	−21.9	−1,761.1 to 1,717.2	0.98			
						
Tofacitinib (n = 5)	−1,977.4	−5,347.3 to 1,392.6	0.248			

CI, confidence interval; COVID-19, corona virus disease 2019; IBD, inflammatory bowel disease; TNF, tumor necrosis factor α.

a Including azathioprine, 6-mercaptopurine, or methotrexate.

## DISCUSSION

We evaluated CMIR at approximately 1–2 months and 6 months after a third mRNA COVID-19 vaccine and found that all patients with IBD regardless of their treatment regimen had measurable responses. We compared CMIR after 2 doses of vaccine (89% in HERCULES study) with CMIR after 3 doses of vaccine, after which 100% of study participants had measurable CMIR, demonstrating benefit of a third dose of mRNA vaccine (21). This has not been evaluated in previous studies in the population with IBD. In addition, our study did not show waning of CMIR at 6 months post 3-dose series. Those on anti-TNF monotherapy had higher CMIR at both time points after the third dose compared with those not on anti-TNF therapy or those on anti-TNF combination therapy with an immunomodulator, which is similar to what we saw after 2 doses of mRNA vaccine (21). Furthermore, those on anti-TNF therapy did not have significant waning of CMIR at 6 months. These findings can reassure patients and providers that even among patients with IBD on immune-modifying therapies such as anti-TNF therapy, immune responses to the vaccine are robust and durable. This is also, to our knowledge, the only study to examine CMIR after a fourth dose of vaccine. Although this subset of patients was smaller, we failed to show a boost in CMIR with a fourth dose of vaccine.

Two previous studies have evaluated CMIR after a third dose of the COVID-19 vaccine (10,22). The COVID-19 vaccine–induced antibody and T-cell responses in immunosuppressed patients with IBD after the third vaccine dose (VIP) study examined antigen-specific T-cell responses 28–49 days after the third vaccine dose (10). Most of the patients with IBD (191/202; 95%) mounted a CMIR. CMIR was similar among COVID-19 infection-naive patients with IBD on thiopurines, infliximab, thiopurine plus infliximab, ustekinumab, or vedolizumab, compared with healthy controls. Infection-naive patients receiving tofacitinib had lower CMIR compared with healthy controls. In those with previous SARS-CoV-2 infection, CMIR against SARS-CoV-2 nucleocapsid mapped epitope pool was significantly lower in patients treated with ustekinumab than in healthy controls.

The systemic and T cell–associated responses to SARS-CoV-2 immunization in gut inflammation (STAR SIGN) study evaluated humoral responses and CMIR 2–16 weeks after receiving the third mRNA vaccine dose among 139 patients on biologics and 110 healthy controls (22). In contrast to the VIP and this study, a significantly higher proportion of those on anti-TNF therapy (15/73, 20.5%) failed to mount an adequate CMIR to the third dose of vaccine, compared with healthy controls (2/100, 2%), as measured by an IFN-γ release assay. There was no significant difference between CMIR in anti-TNF–treated and non–anti-TNF–treated patients with IBD. The VIP study and our study each used ELIspot or Fluorospot to measure CMIR, which has been shown to detect CMIR in immunosuppressed patients more often (27). In addition, these assays measure different aspects of T-cell response. ELISpot/Fluorospot quantifies the number of T cells that respond, whereas the IFN-γ release assay measures the amount of cytokine produced by T cells in response to antigen stimulation.

While the STAR SIGN study found decreased CMIR among those on anti-TNF therapy, the Impact of Biologic and Immunomodulatory Therapy on SARS-CoV-2 Infection and Immunity in Patients with IBD (CLARITY-IBD), Coronavirus Risk Associations and Longitudinal Evaluation-IBD, and HERCULES study groups found that patients with IBD on anti-TNF therapy have similar or augmented CMIR following vaccination with the primary series. The CLARITY-IBD study examined humoral and CMIR to the BNT162b2 and ChAdOx1 nCOV-19 vaccines among patients with IBD treated with infliximab or vedolizumab (9). Approximately one-fifth of patients failed to mount a CMIR in both groups after the primary series. The Coronavirus Risk Associations and Longitudinal Evaluation-IBD study examined CMIR among 303 patients with IBD and found all participants had a T-cell clonal response. Spike-specific T-cell response reduced significantly with age. There were no significant differences in response among those on ustekinumab, vedolizumab, tofacitinib, or steroids. CMIR was augmented among those on anti-TNF therapy after adjusting for age and sex. The HERCULES study found that most of the patients with IBD developed a CMIR after receiving 2 doses of mRNA COVID-19 vaccines. Those on anti-TNF therapy had an augmented CMIR (21). This finding was confirmed in this study where CMIR was augmented in those on anti-TNF monotherapy at both time points after a third dose of COVID-19 vaccine. Those on anti-TNF combination therapy with an immunomodulator did not have an augmented response showing that an augmented CMIR is potentially linked to anti-TNF monotherapy alone. The reason for the augmented response on anti-TNF therapy is unknown but is postulated due to the effects of TNF-α on vaccine-induced humoral and cellular responses (28). TNF alpha supports B-cell maturation and downregulates T-cell expansion. The lack of downregulation would result in an augmented CMIR, and the blunted B-cell maturation would cause lower antibodies in patients on anti-TNF therapy (29,30). These findings would suggest that patient on anti-TNF monotherapy may be at lower risk of severe disease from SARS-CoV2 given the augmented CMIR because T cells can help prevent onset of severe disease for most cases.

The CMIR is responsible for immunologic memory and recognition of viral variants and clearance, generation, and maintenance of antibody responses and is an important component of COVID-19 vaccine efficacy in addition to humoral immunity. The CMIR can protect against viral variants, which may evade humoral immunity (18). The VIP study, STAR SIGN study, and current study demonstrate discordant CMIR and humoral immune responses, suggesting that there is not a strong correlation between antibody and CMIR among patients with IBD (21). Previous studies in healthy controls have also demonstrated discordance in CMIR and antibody response (19). By contrast, the CLARITY-IBD study demonstrated a modest positive correlation between CMIR and antibody concentration among those receiving an mRNA vaccine (9). The discordance in these results demonstrate that both humoral and cell mediated immunity must be evaluated to determine the impact of different immune-modifying therapies on COVID-19 vaccine immunity. Each component only accounts for a part of vaccine-induced immunity.

Both mRNA-1273 (Moderna) and BNT162b2 (Pfizer-BioNTech) elicit a T-helper type1 immune response producing IFN-γ shown in other studies in which only minimal T helper type 2 response was measured (31,32). The presence of CMIR has been shown to protect individuals from infection, even in the absence of neutralizing antibodies (33). SARS-CoV-2–specific cell-mediated immune responses seem to wane more slowly compared with antibody concentrations. In addition, these CMIR recognize conserved viral antigens with less than 15% of T cells recognizing viral mutations sites (19,34). Therefore, CMIR could provide protection from viral variants. All patients with IBD in this study mounted CMIR, which persisted over the vaccine dosing interval. Our study showed slower waning of CMIR compared with antibody concentrations. Clinicians can consider that other benefits of CMIR, such as recognition of at least some viral variants, which have been demonstrated in other studies may offer some additional vaccine protection in patients with IBD.

Our study has several strengths. We evaluated sustained CMIR over multiple time points among patients on stable treatment regimens for at least 2 months. This allowed us to assess the effect of different immunosuppressive medications on vaccine response. We assessed CMIR 6 months after the third dose of vaccine and were able to include a small cohort evaluating immunogenicity after a fourth dose of vaccine. Our study is limited by the lack of a reference healthy control population, inability to evaluate the impact of disease activity on vaccine response, and small representation of certain treatment regimens (tofacitinib, thiopurine monotherapy, and systemic steroids). We were also limited in being unable to evaluate the real-world effectiveness of vaccine in preventing breakthrough infections because the goal of HERCULES was evaluating humoral and cell-mediated immunogenicity of COVID-19 vaccine. Patients in the study were not followed up or asked about breakthrough infections during the study period. Thus, we were unable to evaluate the correlation of breakthrough infections with CMIR or IBD therapy. Evaluating breakthrough infections is a complex issue because the goal of vaccination is preventing severe disease that results in hospitalization. However, some variants are highly contagious and result in higher rates of infections. Furthermore, the impact of IBD therapies on breakthrough infections has been variable in multiple studies (35,36). Future studies should evaluate CMIR and humoral immune response in a larger population of patients in different treatment arms.

In summary, we found that patients with IBD should receive a third dose of an mRNA vaccine to maximize the CMIR and this response is sustained for approximately 6 months. We also confirmed our previous finding that patients on anti-TNF therapy have an augmented CMIR to mRNA COVID-19 vaccines.

## CONFLICTS OF INTEREST

**Guarantor of the article:** Freddy Caldera, DO, MS.

**Specific author contributions:** F.C.: study concept and design, acquisition of data, analysis and interpretation of data, drafting of the manuscript, and critical revision of manuscript; S.R.: drafting of manuscript, critical revision of manuscript; F.A.F.: critical revision of manuscript; B.M.N.: acquisition of data and drafting of manuscript; D.C.: acquisition of data; E.E.Z.: critical revision of manuscript; T.L.S.: acquisition of data and critical revision of manuscript; O.R.R: acquisition of data, critical revision of manuscript; M.A.: acquisition of data, critical revision of manuscript; K.C.: acquisition of data, critical revision of manuscript, analysis and interpretation of data; M.S.H.: study concept and design, acquisition of data, analysis and interpretation of data, drafting of the manuscript, and critical revision of manuscript; K.L.K.: acquisition of data, analysis and interpretation of data, drafting of manuscript, and critical revision of manuscript.

**Financial support:** Takeda Pharmaceuticals, American College of Gastroenterology, and Mayo Clinic.

**Potential competing interests:** F.C. has received research support from Takeda Pharmaceuticals, Janssen, and Novavax. He has been a consultant for Takeda, Arena Pharmaceuticals, GSK, and Celgene. F.A.F. is a consultant for Abbvie Avalo Therapeutics, BMS, Braintree Labs, Fresenius Kabi, GI Reviewers, GSK, IBD Educational Group, Iterative Health, Janssen, Pharmacosmos, Pfizer, Sandoz Immunology, and Sebela. He sits on a DSMB for Lilly Pharmaceuticals. M.S.H. is a consultant for GSK Vaccines and Seqirus and has received research support from Takeda Pharmaceuticals and Dynavax. Dr. Chun is an employee of LabCorp. Dr. Swift has received research support from Pfizer. K.L.K. is the Andrew A. and Mary S. Sugg Professor of Cancer Research and reports consulting fees from Leidos, Antigen Express, and Affyimmune; grant and other funding to Mayo Clinic from Marker Therapeutics, Macrogenics, Bolt Therapeutics and Tallac; royalties from Marker Therapeutics; and stocks from Kiromic, Inc.

**Study Highlights**

**WHAT IS KNOWN**

✓ Patients with inflammatory bowel disease mount a cell-mediated immune response after a second and third dose of COVID-19 vaccine.

**WHAT IS NEW HERE**

✓ A third dose of a COVID-19 vaccine boosts cell-mediated immune response, and the response is sustained for 6 months. There is no significant boosting after a fourth dose. Those on anti–tumor necrosis factor monotherapy have an augmented response compared with those not on anti–tumor necrosis factor monotherapy.

## Supplementary Material

**Figure:**
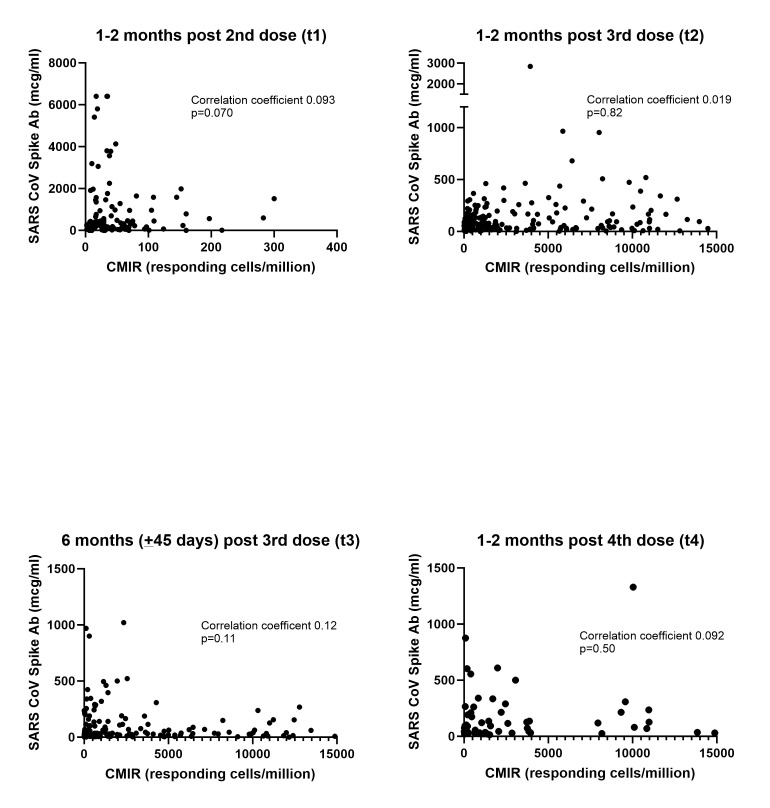

